# *Plasmodium* spp. Infections Among Mbalmayo Inhabitants of Central Region in Cameroon: Discrepancies Between Rapid Diagnostic Tests and Molecular Methods

**DOI:** 10.3390/pathogens14050462

**Published:** 2025-05-09

**Authors:** Lidia Stopyra, Wanesa Wilczyńska, Daria Kołodziej, Assamba Noel, Krzysztof Korzeniewski

**Affiliations:** 1 Clinic of Infectious and Tropical Diseases, Andrzej Frycz Modrzewski University, 30-705 Kraków, Poland; lidiastopyra@gmail.com; 2 Department of Epidemiology and Tropical Medicine, Military Institute of Medicine—National Research Institute, 04-141 Warsaw, Poland; wwilczynska@wim.mil.pl (W.W.); dkolodziej@wim.mil.pl (D.K.); 3 Mbalmayo District Hospital, P9, Oyack II, Nyong-et-Soo, Centre 237, Mbalmayo NT-006, Cameroon; nassamba@yahoo.com

**Keywords:** malaria, *Plasmodium*, mRDT, RT-PCR, Cameroon

## Abstract

Malaria remains a major public health threat in Cameroon, with an estimated 3 million new cases of *Plasmodium* spp. infections reported each year. The aim of this study was to assess the occurrence of *Plasmodium* infections in Cameroon in a group of symptomatic and asymptomatic individuals, residents of the town of Mbalmayo, located in the Central Region of Cameroon. Screening was conducted in December 2024 at the Mbalayo District Hospital. This study involved a total of 93 people aged between 1 and 70 years old, who voluntarily agreed to have their blood samples taken and tested for malaria. As part of this study, the demographic variables of the participants were taken, malaria rapid diagnostic tests (mRDTs) were performed, and blood samples were applied to the Whatman FTA cards for further real-time PCR diagnostics. The occurrence of *Plasmodium* infections in the residents of Mbalmayo differed depending on the diagnostic method used (30.1% with mRDT vs. 60.2% when RT-PCR assays were performed). A total of 55 malaria cases were found to be caused by *P. falciparum*, while one case was found to be caused by *P. vivax*. Nearly half of the study participants exhibited no signs or symptoms of malaria, whereas 35.7% reported fever, 17.9% respiratory symptoms, and 10.7% gastrointestinal symptoms. The prevalence of malaria remains high in populations inhabiting the Central Region in Cameroon. *P. falciparum* is the dominant species in the region. A considerable proportion of infected individuals are asymptomatic, which supports the finding that asymptomatic carriers play a critical role in disease transmission. The differences between the results depending on the diagnostic method used (mRDT vs. RT-PCR) suggest that there is a need to use a combination of different methods for the identification of malaria, especially in cases of low parasitemia.

## 1. Introduction

Cameroon is a country lying in the western parts of Sub-Saharan Africa, with a population of over 28 million people. The country’s gross domestic product (GDP) per capita is several times lower than the global average, and its human capital index (HCI) is 0.4 [1,2]. Cameroon has a high burden of infectious and invasive diseases. Poor living standards, a low level of education, and its tropical climate have been identified as significant contributors to the transmission of infectious diseases, including malaria.

In fact, malaria remains one of the most important health problems in the country. Cameroon is one of 20 countries that carry 70% of the global malaria burden [3,4]. Each year, the country reports more than 3 million new cases of *Plasmodium* spp. infections, and malaria remains the leading cause of hospital admissions in Cameroon [5]. According to the data released by the Cameroon Ministry of Public Health, malaria accounts for 30–35% of the general mortality and 67% of childhood mortality per year [6].

Although all regions of Cameroon are at high risk of malaria transmission, the distribution of malaria is uneven across the country, as it is largely influenced by specific environmental factors. The data from the Demographic and Health Survey (DHS) as well as from the Malaria Indicator Survey (MIS) indicated vegetation and altitude as important predictors of the geographical distribution of malaria in Cameroon [7]. Malaria transmission tends to be more intense and is year-round in forests, humid savannahs, and coastal settings, whereas seasonal transmission occurs in the mountains and the dry Sahel savannah. Malaria transmission in Cameroon is primarily mediated by species belonging to the *Anopheles gambiae*, *Anopheles funestus*, and *Anopheles nili* complexes, which are considered the main vectors of the disease. In certain regions of the country, so-called secondary vectors, such as *Anopheles moucheti* and *Anopheles paludis*, have also been identified, which may locally contribute to malaria transmission, especially where environmental conditions are favorable. In settings with the highest malaria transmission, each person may be exposed to as many as 100 mosquito bites per month. It is estimated that more than 70% of the country’s population lives in areas where malaria transmission is particularly intense [8,9,10].

Malaria prevention in Cameroon mostly relies on vector control, which is achieved through the distribution of long-lasting insecticidal nets (LLINs). Since 2020, Cameroon has received considerable support from international organizations such as the World Health Organization and the Global Fund, which provide free bed nets to all households [10]. These interventions contributed to a considerable reduction in malaria morbidity and mortality (from 13,000 to 6000 deaths annually) between 2000 and 2015 [11]. Unfortunately, the rate of decline in malaria morbidity has slowed in recent years. According to the 2022 data from the National Institute of Statistics (NIS), more than 70% of households in Cameroon have at least one LLIN, but only 54% of households use it on a regular basis (bed nets are mostly used by pregnant women and children) [12]. In addition, some vectors are becoming increasingly resistant to insecticides used for malaria prevention [13]. In addition to the growing resistance of mosquito vectors to insecticides, antimalarial drug resistance is an emerging concern in Cameroon. The country has reported cases of resistance to commonly used antimalarial drugs, particularly to chloroquine and sulfadoxine–pyrimethamine, which has led to changes in treatment guidelines over the past two decades [5].

The malaria control program in Cameroon faces numerous challenges, such as the previously mentioned growing resistance of vectors to insecticides and an increasing number of multi-drug-resistant parasites in the region, but there are many more other factors that make it increasingly difficult to control malaria in the region, including an increase in the rate of urbanization, mass migration, as well as poor sanitation and wastewater management [1].

In view of the above, the aim of the present study was to assess the occurrence of symptomatic and asymptomatic malaria in Cameroon in a study involving a sample of residents from Mbalmayo, a town in the Nyong-et-Soo Department, the Central Region of Cameroon. This study involved both symptomatic patients and asymptomatic carriers, the latter serving as an important parasite reservoir responsible for maintaining malaria transmission.

## 2. Materials and Methods

### 2.1. Study Group

**Figure 1:**
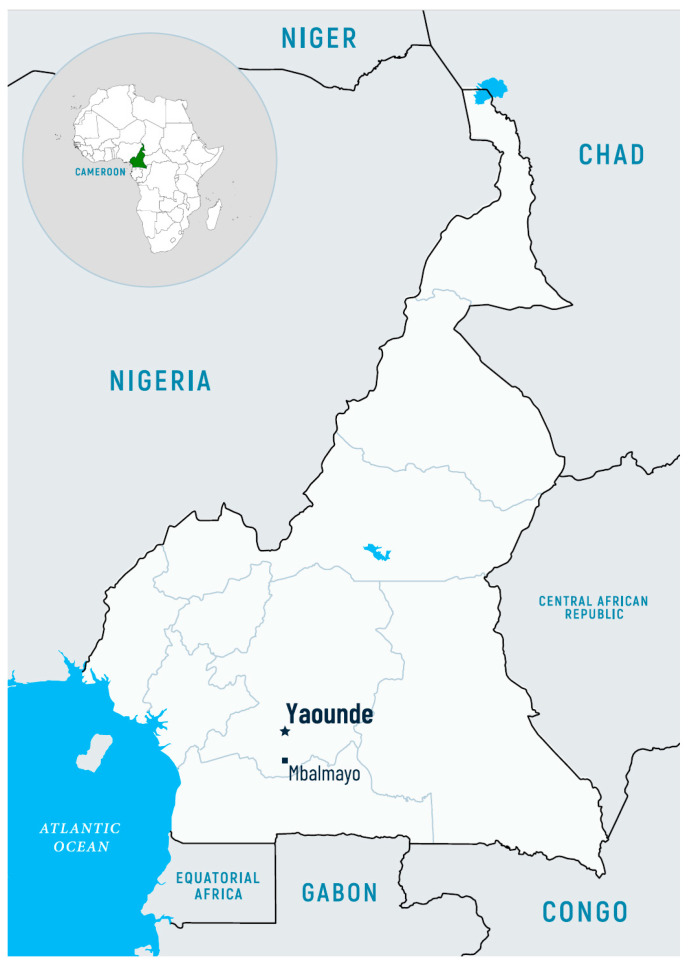
Map of Cameroon with the location of Mbalmayo.

Malaria screening was conducted in December 2024 at the Hospital De District De Mbalmayo, which is located in Mbalamayo (location 3°31′ N 11°30′ E), a town inhabited by 80,000 people. The town lies in the Nyong-et-Soo Department in Central-Southern Cameroon (Figure 1).

The region inhabited by the participants of the present study features a large variety of ecosystems, ranging from tropical rainforests to humid savannah. There are numerous stagnant or slow-moving water bodies in the region, which form suitable habitats for the *Anopheles* mosquito, a vector of malaria transmission. In addition, intense and prolonged rainfall (1500 mm annually) favors the development of mosquito larvae. South Cameroon is holoendemic for malaria; the region has an exceptionally high malaria transmission occurring year-round. Most of the local inhabitants are engaged in agriculture and, therefore, have frequent contact with stagnant water bodies (both as part of their work and in their everyday lives). This greatly increases their risk of exposure to malaria vectors, thus increasing the risk of acquiring the disease [11]. Participation in this study was voluntary.

A total of 93 people aged between 1 and 70 years old were enrolled in this study, regardless of their sex, age, presence/absence of malaria signs and symptoms. Each participant provided written informed consent to participate in this study. For participants under 18 years old, the consent was provided by their parents or legal guardians. In the age categories, the participants were grouped according to their age in full years, with individuals aged 14 years and 3 months, for example, being categorized as 15 years old. This classification was applied consistently for all participants.

### 2.2. Sample Collection

#### 2.2.1. Malaria Rapid Diagnostic Tests (mRDTs)

The study participants reported to the Hospital De District De Mbalmayo, where medical personnel recorded their demographic variables (age and sex), conducted patient interviews, and recorded any symptoms reported by the patients (fever, respiratory symptoms, and gastroenterological symptoms). Next, venous blood samples were collected from each participant, and malaria rapid diagnostic tests (mRDTs) were conducted. The tests for malaria were conducted using a lateral flow chromatographic immunoassay, NADAL^®^ Malaria Pf/Pan Ag 4 species (nal von minden GmbH, Regensburg, Germany). This test can be used for the simultaneous, qualitative detection and differentiation of HRP II (histidine-rich protein II), specific to *P. falciparum*, and pLDH (lactate dehydrogenase), specific to *Plasmodium* species. According to the information provided by the manufacturer, the test’s sensitivity and specificity were 99.1%. All patients with *Plasmodium* infection detected by mRDTs were treated with antimalarial therapy.

#### 2.2.2. Molecular Diagnostics (RT-PCR)

Approximately 200–300 µL of the venous blood (from the same samples as were first used for the mRDTs) was applied onto Whatman FTA cards. The cards were left to dry, and next, they were put in tightly sealed foil packages with a moisture absorber. The dried blood spots preserved on the Whatman FTA cards were then transported to the Department of Epidemiology and Tropical Medicine of the Military Institute of Medicine, the National Research Institute in Poland, where molecular tests were performed. Four 2 mm discs punched out from the Whatman FTA cards with a Harris Uni-Core punch (Qiagen, Hilden, Germany) were used to isolate the genetic material. The DNA was extracted using the Sherlock AX Kit (A&A Biotechnology, Gdańsk, Poland) in line with the manufacturer’s instructions, and the final elution volume was 100 µL. The Sherlock AX Kit is a test for the manual isolation of genomic DNA, which works on the principle of nucleic acid absorption on ion-exchange membranes, combined with DNA precipitation with isopropanol. The extracted samples were stored at a temperature of −20 °C for further tests. The Bosphore^®^ Malaria Genotyping Kit v1 (Anatolia Geneworks, Istanbul, Turkey) targeting genes encoding the *Plasmodium* spp. 18S rRNA was used to perform RT-PCRs. This assay can be used for the detection and differentiation of four different *Plasmodium* species: *P. falciparum*, *P. malariae*, *P. ovale*, and *P. vivax*. The RT-PCRs were run on an AriaMx RT-PCR system (Agilent Technologies, Santa Clara, CA, USA), following the thermal protocol set by the system’s manufacturer (Table 1).

### 2.3. Statistical Analysis

The statistical analyses were conducted using StatSoft Inc.’s STATISTICA version 12.0 software (StatSoft Polska Sp. z o.o., Kraków, Poland), accessed on 27 February 2025 (www.statsoft.pl), along with an Excel spreadsheet (Microsoft Corporation, Redmond, WA, USA). The significance of the differences between the groups of *Plasmodium*-infected vs. non-infected individuals was determined with the Mann–Whitney U test or chi-square. In all calculations, the threshold for statistical significance was set at *p* = 0.05.

### 2.4. Ethical Approval

This research project was approved by the Committee on Bioethics at the District Medical Chamber in Kraków, Poland (Decision No. 145/KBL/OIL/2024; 12 December 2024). The screening in the Cameroonian community was performed with the written consent of every patient and with the assistance of medical personnel working at the Hospital De District De Mbalmayo.

## 3. Results

A total of 93 people were enrolled and tested for malaria by RDTs and PCR tests, of which 47.3% were women and 52.7% were men. Most of the study participants did not report or exhibit any disease signs and symptoms (61.3%), while 28% of the participants reported fever, 12.9% presented with respiratory symptoms, and 7.5% presented with gastrointestinal symptoms. The mean age of the study participants was 20.3 years; children under the age of 15 years accounted for 55.9% of all the participants. The demographic variables of the study participants are presented in Table 2.

Malaria rapid diagnostic tests (mRDTs) showed that a total of 28 participants (30.1%) were infected with *Plasmodium* spp.; HRP II (which is indicative of *P. falciparum* infection) was detected in 19 of the infected individuals; pLDH (indicative of *Plasmodium* spp.) was found in 6 of the infected participants; and both HRP II and pLDH were detected in 3 people. The RT-PCR tests showed a two-fold higher positive case rate (60.2%) compared with the mRDTs. The statistical analysis confirmed that the differences in the results between the two testing methods were statistically significant (*p* < 0.05). Two mRDT-positive results (one positive for *P. falciparum* and the other for Pan (*Plasmodium* spp.)) were not confirmed by the RT-PCR assay. All other *P. falciparum*-positive mRDT results were confirmed by molecular methods. Also, molecular tests detected the genetic material of *P. falciparum* in five mRDTs that were positive for Pan, including one case of *P. falciparum* and *P. vivax* co-infection. Three mRDTs, which were positive both for Pan and *P. falciparum*, were found to be *P. falciparum*-positive only. The malaria infection rate was found to be higher in men compared with women, but the difference in this respect was not statistically significant (*p* = 0.2899) (Table 3). Most malaria cases were observed in participants aged < 15 yrs. and >50 yrs. (when RT-PCR methods were applied). Furthermore, a statistically significant difference was found between the rates of symptomatic and asymptomatic malaria, at 83.3% and 45.6%, respectively (*p* < 0.05). A total of 35.7% of malaria-infected individuals reported fever, 17.9% had respiratory symptoms, and 10.7% had gastrointestinal symptoms.

## 4. Discussion

Although most regions in Cameroon experience intense, year-round malaria transmission, certain areas—such as the highlands and the Sahel savannah—are characterized by more seasonal patterns, influenced by altitude and rainfall variability. However, due to the underreporting of cases and limited access to healthcare services, it is difficult to estimate the actual malaria morbidity in many regions. Studies that have been conducted locally demonstrate that the incidence of malaria in Cameroon ranges between 8.8% and 64.7%, depending on the region, study sample composition, and methodology applied [12,13,14,15,16,17,18,19,20,21,22,23,24]. The results of the present study are in line with the findings of some researchers [19,22,23]. It needs to be highlighted, however, that our results differ significantly depending on the diagnostic method used (mRDT vs. molecular methods). The results of mRDTs showed an overall malaria prevalence of 30.1%, whereas the results of molecular tests showed that the prevalence of infections caused by *Plasmodium* spp. was twice as high (60.2%). Such significant differences between the two testing methods are worrying and point to the fact that the true burden of malaria in Cameroon may be underestimated if mRDT results are not verified by more precise molecular methods. Other authors have also pointed out considerable discrepancies between the results of mRDTs and RT-PCR assays. This study identified two types of *Plasmodium* infections: *P. falciparum* and *P. vivax* infections. However, *P. falciparum* infections were predominant. This is not surprising, considering the fact that *P. falciparum* has been documented to be responsible for over 95% of all malaria cases in Cameroon [25]. The RDTs identified six cases of Pan infections and three cases of Pf/Pan co-infections. The presence of a double band (Pf/Pan) on the test strip may be indicative of higher parasitemia, i.e., in more severe cases. A study conducted by Djoufounna et al. [26] found that the mean density of *Plasmodium* was higher in the case of the presence of a double band in an mRDT test, which suggests that higher parasitemia increases the odds of detecting both antigens (Pf and Pan). One mRDT Pan-positive result was not confirmed by RT-PCR methods, which could have been due to a low parasite density or the presence of other factors impacting the accuracy of the test result [26]. It should also be noted that the concentration of *Plasmodium falciparum* lactate dehydrogenase (pfLDH), which is detected by some RDTs, increases more slowly and is typically lower than HRP2 levels. Therefore, in cases of recent or low-density infections, pfLDH-based detection may result in false-negative results. Conversely, the longer the infection circulates, the higher the pfLDH concentration becomes, which may improve the sensitivity of tests based on this antigen. Five samples that were positive for *P. falciparum* by RT-PCR, and showed by mRDT only the Pan band without the Pf band, may have been associated with the deletion of the Pfhrp2 gene in *P. falciparum*. According to the literature, *P. falciparum* with Pfhrp2 gene deletions is found in many malaria-endemic African countries, and its prevalence may reach as high as 60% in some regions [27,28,29]. *P. falciparum* with Pfhrp2 gene deletions has been documented to widely occur in Cameroon as well [26,30,31,32], which could be one of the reasons for the absence of the Pf band (in the presence of the Pan band) in the mRDTs, which were positive for *P. falciparum*, and could also explain the false-negative results. As has been mentioned before, the substantial differences in the sensitivity of the two testing methods used suggest that mRDTs are not sensitive enough to detect all malaria infections, especially in cases of low parasitemia [33,34,35]. This finding supports the necessity to combine multiple diagnostic tools in malaria screening to achieve more accurate and reliable results. Such differences in test results may affect malaria control strategies, especially in areas where access to advanced diagnostic methods is limited. Malaria RDTs are quick and easy to use; however, they have variable specificity and sensitivity. Therefore, relying on the results of mRDTs alone can lead to errors in the assessment of the epidemiological situation, impacting the effective planning of malaria control interventions [26,30,31,32]. Effective malaria surveillance needs well-integrated quality control procedures assessing the sensitivity and specificity of mRDTs [26].

The present study found one case of *P. vivax* infection. However, 98% of the identified malaria cases were caused by *P. falciparum*. Other authors have demonstrated that two other *Plasmodium* species (*P. malariae* and *P. ovale*) are also circulating in Cameroon, but the results of our study found no malaria cases caused by either of the two species. According to the available literature, *P. falciparum* is responsible for the vast majority of malaria cases in the area where malaria screening was performed [25], whereas *P. vivax* infections are primarily found in the southern regions of the country [36,37], which is consistent with the results of our study.

The latest research results suggest that young children are most affected by malaria and that malaria-infected children usually exhibit significantly higher parasitemia compared with patients from older age groups. Children under the age of five years are the most vulnerable to malaria because they have not yet acquired natural immunity. In high-transmission settings, multiple exposures to *Plasmodium* species at an early age result in the development of natural immunity to malaria, which reduces the risk of re-infection at an older age [19,26]. The results of our study support the findings that suggest that malaria is most prevalent in the pediatric age group and that both the malaria prevalence and infection intensity decrease with age. In the group of patients aged 50 years or older, the RT-PCR assays showed a nearly eight-fold increase in the number of positive cases compared with the results obtained from the mRDTs. It is a well-established fact that people’s immunity decreases with age; as a result, older individuals become more susceptible to malaria, although in their case, the disease often takes the form of a chronic, low-density *Plasmodium* infection, which can be missed in screening if less-sensitive diagnostic tools (such as mRDTs) are used [38,39].

The present study found that *Plasmodium* infections were more prevalent in men compared with women (65.3% vs. 54.5%); however, the difference was not statistically significant (*p* = 0.2899). The difference in the malaria prevalence between males and females may be explained by the type of work men typically perform in the study area, i.e., men tend to work outside more often than women and are, therefore, at a higher risk of exposure to mosquito bites (the vectors of infection) [15,20,22]. Meanwhile, there are some studies from Cameroon that suggest that the prevalence of malaria is higher among women than men. A possible explanation for this might be the fact that women from some communities often work/perform their chores outside at times when mosquitoes are most active, i.e., at dusk and dawn, and are, therefore, more susceptible to mosquito bites [40]. Other studies, however, do not support these findings and suggest, instead, that gender itself is not a risk factor for malaria acquisition [26,41].

This study found a statistically significant difference between the rates of symptomatic and asymptomatic malaria cases (*p* = 0.0002). As much as 47% of the study participants who tested positive for malaria were asymptomatic, which is consistent with previous reports pointing to a high prevalence of asymptomatic carriage in many malaria-endemic settings. The existence of asymptomatic malaria reservoirs poses a challenge that may hinder the elimination of malaria. Asymptomatic carriers can harbor transmissible gametocytes and contribute to sustaining malaria transmission, especially in high-endemic settings. The prevalence of asymptomatic malaria is comparable to that found in other studies in sub-Saharan Africa, where asymptomatic carriage has been found to be a significant contributor to malaria transmission. For example, in studies conducted in Nigeria and Cameroon, asymptomatic malaria rates ranged from 19% to 64%, depending on the region and diagnostic methods used [42,43]. Similarly, in Gabon, 6.2% of adults in rural populations were asymptomatically infected, with even higher rates in certain villages [44]. In the Central African Republic, 32.4% of children in Bantu communities and 40.6% in BaAka Pygmy communities were found to be asymptomatically infected [45]. These findings underscore the need for a comprehensive understanding of asymptomatic carriage across the African continent, as it poses a critical challenge to malaria elimination strategies.

These individuals often go undetected by routine diagnostic tools such as RDTs or microscopy, thus remaining untreated and forming a hidden reservoir [19,41]. In high-transmission settings, i.e., in areas where people commonly become reinfected with *Plasmodium* species, asymptomatic malaria cases are more often found in adults than in children. This finding has been supported by the results of the present study, which show that the mean age of asymptomatic malaria carriers was 22.7 years, while the mean age of symptomatic individuals was 14.9 years. Based on these results, it can be concluded that symptomatic malaria is more common in children than in adults [23,41].

Fever is the most characteristic sign of malaria. It typically has an acute onset and a cyclical pattern, with fever recurring every 48 or 72 h, depending on the parasitic species that caused the infection (the fever pattern in *P. falciparum* malaria is often irregular). Other common signs and symptoms of malaria include chills and general weakness. As the disease progresses, some patients may develop an extremely high temperature and will need continuous monitoring. Apart from the characteristic fever, some malarial patients can present with respiratory symptoms, with tachypnoea (rapid breathing) and cough being the most common. Respiratory symptoms are the body’s response to parasitic invasion, but they can also be indicative of systemic inflammation or pneumonia, especially in more severe cases. Malaria can also manifest with gastrointestinal signs and symptoms, such as nausea or vomiting. These symptoms may lead to dehydration (especially in small children), impacting the patient’s general condition and potentially leading to more serious complications. Other common signs and symptoms of malaria may also include abdominal pain and a loss of appetite (suggesting invasion of the liver or spleen by parasites) [46]. In this study, fever was reported by 35.7% of the infected individuals, respiratory symptoms by 17.9%, and gastrointestinal symptoms by 10.7%. In our study, gastrointestinal symptoms were only seen in patients under 9 years old, which suggests that children are more susceptible to gastrointestinal manifestations of malaria than adults. It needs to be stressed, however, that these co-occurring symptoms might be a sign of infections other than malaria. In fact, Cameroon is endemic for many infectious diseases, which have similar signs and symptoms to malaria. A differential diagnosis should consider other infectious diseases as well, especially if signs and symptoms are non-specific or in patients living in areas that are endemic for various communicable diseases [42,43,47,48].

Despite significant efforts to combat malaria, the disease remains one of the major health threats in many African countries. Successful malaria control requires decisive and coordinated interventions, but the fight against the disease is heavily impacted by the antimicrobial resistance of *Plasmodium* species, a growing resistance of vectors to insecticides, and limited financial resources [49,50]. In Cameroon, the progress toward malaria control has slowed in recent years. To return to the right track, it is necessary to not only tighten the efforts against malaria but also engage local communities more strongly. Inconsistent use of bed nets, a lack of interventions whose aim would be to eliminate vectors’ breeding sites, and relying on self-treatment rather than seeking professional medical aid are the most important factors behind the increase in malaria transmission rates, increased reproduction of vectors, and growing antimicrobial resistance in Cameroon [23]. In Cameroon, the resistance of *P. falciparum* to antimalarial drugs poses an increasing challenge for malaria control. Chloroquine and sulfadoxine–pyrimethamine are no longer recommended for treatment due to widespread resistance. The national malaria treatment guidelines currently recommend artemisinin-based combination therapies (ACTs), such as artemether–lumefantrine, as the first-line treatment for uncomplicated malaria, and intravenous artesunate for severe cases [49]. However, molecular surveillance has identified the emergence of mutations associated with reduced sensitivity to artemisinin in some regions of Cameroon, raising concerns about future drug efficacy [51].

In the study area of Mbalmayo, malaria case management is largely based on national protocols. Most public health facilities utilize rapid diagnostic tests (RDTs) as the primary diagnostic tool, with confirmatory microscopy or molecular tests being used less frequently due to cost and availability. ACTs are typically provided free of charge in government-run clinics. However, limited access to healthcare in rural communities, occasional shortages of antimalarial drugs, and the use of traditional medicine practices may delay appropriate treatment and contribute to ongoing transmission [52]. Strengthening community education, ensuring uninterrupted drug supply chains, and integrating more sensitive diagnostic tools (e.g., molecular diagnostics) into selected use cases—such as targeted screening in pregnant women or pre-elimination settings—may contribute to improving malaria case management. However, given the high-transmission setting and resource constraints in Cameroon, such approaches must be carefully evaluated for feasibility and cost-effectiveness before widespread implementation. A potential breakthrough in the fight against malaria will require coordinated interventions, which combine the implementation of an effective health policy, the education of local communities, and the introduction of novel medical technologies. Only such an integrated policy will accelerate the efforts toward global malaria elimination.

In particular, the findings of this study highlight the importance of enhancing diagnostic capabilities through more sensitive and specific diagnostic tools, such as RT-PCR, to detect both symptomatic and asymptomatic malaria cases. This approach could significantly improve the early detection and treatment of infections, thereby reducing transmission. Additionally, addressing the vector control challenges by focusing on both primary and secondary malaria vectors will be crucial for enhancing the impact of current prevention strategies. Moreover, increasing awareness and providing education at the community level are essential for encouraging the uptake of preventive measures, such as insecticide-treated nets and timely treatment. By combining these efforts with robust surveillance systems, the national malaria program in Cameroon can better target interventions, reduce the malaria burden, and contribute to the long-term goal of malaria elimination.

## 5. Limitations of This Study

This study had several limitations, which could have impacted the results. First, the participants were not categorized into separate groups according to their general health status or the presence or absence of other conditions that are regionally endemic and which could potentially give similar signs and symptoms as malaria. The hemoglobin (Hb) concentration is one of the major clinical parameters that is used to confirm symptomatic malaria cases. In this study, however, only patients who tested positive in the mRDTs had their Hb concentration measured (the mean Hb concentration in this group was found to be 9.9 g/dL). An important limitation was the small sample size. Furthermore, sampling was conducted at a healthcare facility rather than in the community, meaning that only individuals seeking treatment were surveyed, which may not reflect the actual prevalence in the general population. Additionally, due to the cross-sectional nature of this study, participants were not followed up over time. This limited the ability to distinguish truly asymptomatic cases from pre-symptomatic ones, as some individuals classified as asymptomatic may have developed symptoms after the initial assessment. Despite these limitations, the results of this study provide valuable preliminary data on the prevalence of malaria in the Central Region of Cameroon.

## 6. Conclusions

This study found that the occurrence of malaria among the residents of Mbalmayo was high, and *Plasmodium falciparum* is the dominant malaria species in the Central Region of Cameroon. Nearly 50% of *Plasmodium*-infected individuals were asymptomatic, which supports the fact that asymptomatic carriers play a critical role in disease transmission. The identification of asymptomatic cases should be a key component in malaria elimination efforts. The differences between the test results (mRDT vs. RT-PCR) indicate that there is a need to utilize a combination of different diagnostic methods for malaria-screening purposes (especially in cases of low parasitemia). Such an approach will improve malaria detection rates and contribute to successful malaria control.

## Figures and Tables

**Table 1:** Thermal protocol for the Bosphore^®^v1 Malaria Genotyping Kit used in this study.

Phase	Cycles	Temperature [°C]	Time [min]
Polymerase activation	1	95	14:30
Denaturation	50	97	00:30
Annealing (data collection)		60	01:00
Hold	1	32	05:00

**Table 2:** Plasmodium infections detected with mRDT vs. RT-PCR among Mbalmayo inhabitants (*n* = 93).

Variable		mRDT				RT-PCR		
		Pf	Pan	Pf + Pan	Total	*P. falciparum*	*P. vivax*	Total
Total	*n* (%)	*n* (%)	*n* (%)	*n* (%)	*n* (%)	*n* (%)	*n* (%)	*n* (%)
	93 (100%)	19 (20.4%)	6 (6.5%)	3 (3.2%)	28 (30.1%)	55 (59.1%)	1 (1.1%)	56 (60.2%)
Sex								
Female	44 (47.3%)	7 (15.9%)	2 (4.5%)	2 (4.5%)	11 (25.0%)	24 (54.5%)	0 (0.0%)	24 (54.5%)
Male	49 (52.7%)	12 (24.5%)	4 (8.2%)	1 (2.0%)	17 (34.7%)	31 (63.3%)	1 (2.0%)	32 (65.3%)
Age								
<5	35 (37.6%)	8 (22.9%)	3 (8.6%)	1 (2.9%)	12 (34.3%)	22 (62.9%)	1 (2.9%)	23 (65.7%)
5–14	17 (18.3%)	7 (41.2%)	0 (0.0%)	2 (11.8%)	9 (52.9%)	12 (70.6%)	0 (0.0%)	12 (70.6%)
15–29	14 (15.1%)	2 (14.3%)	2 (14.3%)	0 (0.0%)	4 (28.6%)	7 (50%)	0 (0.0%)	7 (50%)
30–49	14 (15.1%)	1 (7.1%)	1 (7.1%)	0 (0.0%)	2 (14.3%)	6 (42.9%)	0 (0.0%)	6 (42.9%)
≥50	13 (14.0%)	1 (7.7%)	0 (0.0%)	0 (0.0%)	1 (7.7%)	8 (61.5%)	0 (0.0%)	8 (61.5%)
Clinical phenotype								
Symptoms	36 (38.7%)	18 (50.0%)	2 (5.6%)	3 (8.3%)	23 (63.9%)	30 (83.3%)	0 (0.0%)	30 (83.3%)
Fever	26 (28.0%)	9 (34.6%)	2 (7.7%)	2 (7.7%)	13 (50.0%)	20 (76.9%)	0 (0.0%)	20 (76.9%)
Respiratory	12 (12.9%)	8 (66.7%)	0 (0.0%)	1 (8.3%)	9 (75.0%)	10 (83.3%)	0 (0.0%)	10 (83.3%)
Gastroenterological	7 (7.5%)	4 (57.1%)	0 (0.0%)	2 (28.6%)	6 (85.7%)	5 (71.4%)	0 (0.0%)	5 (71.4%)
No symptoms	57 (61.3%)	1 (1.8%)	4 (7.0%)	0 (0.0%)	5 (8.8%)	25 (43.9%)	1 (1.8%)	26 (45.6%)

Pf—*P. falciparum* infection; Pan—*Plasmodium* species infection. The “symptoms” variable captured the presence or absence of common clinical manifestations associated with malaria, which included fever, respiratory symptoms (e.g., cough and difficulty breathing), and gastrointestinal symptoms (e.g., nausea, vomiting, and diarrhea). These three specific symptoms were grouped together under the “symptoms” variable to provide a comprehensive view of the clinical presentation in the study participants.

**Table 3:** Comparative analysis of variables between infected and non-infected Mbalmayo inhabitants (*n* = 93).

Variable	Infected (*n* = 56)	Non-Infected (*n* = 37)	Total (*n* = 93)	*p*-Value
Gender				0.2899 ^1^
Female	24 (42.9%)	20 (54.1%)	44 (47.3%)	
Male	32 (57.1%)	17 (45.9%)	49 (52.7%)	
Age				0.2328 ^2^
Mean (SD)	18.5 (21.5)	23.0 (21.2)	20.3 (21.5)	
Range	1–70	1–70	1–70	
Median	8.5	23	10	
Clinical phenotype				0.0002 ^1^
Symptoms	30 (53.6%)	6 (16.2%)	36 (38.7%)	
Asymptomatic infection	26 (46.4%)	31 (83.8%)	57 (61.3%)	

^1^ Chi-square test; ^2^ Mann–Whitney U test.

## Data Availability

The data presented in this study are available upon request from the corresponding author.
